# Rapid liver graft implantation in canine: A preliminary study

**DOI:** 10.1016/j.sopen.2024.10.006

**Published:** 2024-10-29

**Authors:** Jie Hao, Jia-Wei Yu, Jing-Wen Xiao, Lin-Biao Xiang, Rong Peng, Jia-Qi Quan, Ya-Xu Dong, En-Hui Li, Juan-Juan Wang, Lu Ren, Yong Wan, Hong-Ke Zhang, Yi Lv, Qiang Lu

**Affiliations:** a National Local Joint Engineering Research Center for Precision Surgery and Regenerative Medicine, The First Affiliated Hospital of Xi'an Jiaotong University, Xi'an, Shaanxi, China; b Department of Geriatric Surgery, The First Affiliated Hospital of Xi'an Jiaotong University, Xi'an, Shaanxi, China; c Department of International Medical Center, The First Affiliated Hospital of Xi'an Jiaotong University, Xi'an, Shaanxi, China; d Department of Pediatric Surgery, The First Affiliated Hospital of Xi'an Jiaotong University, Xi'an, Shaanxi, China; e Department of Hepatobiliary Surgery, The First Affiliated Hospital of Xi'an Jiaotong University, Xi'an, Shaanxi, China; f National Local Joint Engineering Research Center for Precision Surgery and Regenerative Medicine, The First Affiliated Hospital of Xi'an Jiaotong University, Xi'an, Shaanxi, China

**Keywords:** Revascularization, Orthotopic liver transplantation, Anhepatic time, Vascular occlusion time

## Abstract

**Background:**

The current method for liver graft implantation during the anhepatic phase is complex. Therefore, this study aimed to introduce a modified orthotopic liver transplantation (OLT) technique with major vascular reconstruction using cuff technique to simplify the process of liver graft implantation during the anhepatic phase.

**Methods:**

Twenty-four canines were randomly assigned to two groups: the modified orthotopic liver transplantation group (M-OLT, *n* = 12) and the control group (n = 12). All animals were randomly assigned to the donor or recipient groups. The recipients received orthotopic liver transplantation using a modified technique in the M-OLT group, and OLT using traditional implantation technique without venovenous bypass was performed in the control group. The donor and recipient characteristics were compared between the two groups. Vascular anastomotic patency was evaluated using angiography immediately and one week after surgery.

**Results:**

All recipients underwent successful liver transplantation. There were no significant differences between the two groups in terms of sex, body weight, or cold ischemia time of the donor liver. However, recipients in the M-OLT group had a shorter operation time, less intraoperative blood loss, shorter anhepatic phase, shorter vascular occlusion time, and shorter warm ischemia time than that in the control group (all *p* < 0.05). No anastomotic leakage or stenosis was detected in either group after liver transplantation. One recipient in the M-OLT group and three in the control group died within one week of transplantation.

**Conclusions:**

This modified technique is safe and feasible for canine liver transplantation and can significantly simplify liver graft implantation procedures during the anhepatic period.

## Introduction

Liver transplantation has been an effective treatment for end-stage liver disease since first described by Starzl et al. in 1963 ([1], [2], [3], [4]). The native liver along with the retrohepatic inferior vena cava (IVC) is removed by the classic orthotopic liver transplantation (OLT) technique, followed by the implantation of the whole donor liver graft with the interposed donor IVC. Despite the manual suture technique being complex and time-consuming, it remains the standard method for liver graft implantation in OLT. However, this will lead to long operation time and warm ischemic time of the liver graft, especially in laparoscopic liver transplantation (5). Previous studies have indicated that shorter liver graft implantation provided significantly better post-transplant outcomes ([6], [7], [8]). The implantation time of the liver graft depends on the duration of the major vascular reconstruction. The cuff technique has been considered as a simple and fast method for major vascular reconstruction ([9], [10], [11]). Based on this, this study aimed to introduce a modified OLT method using cuff technique for rapid liver graft implantation period in canine liver transplantation.

## Materials and methods

### Animals

An estimated 12 liver transplantation events would be needed to provide 90 % power for statistical analysis. Twenty-four beagles (18–24 months of age; 11 males and 13 females; weighing 14–23 kg) were used in this study, and those canines were provided by the Experimental Animal Institute of Xi'an Jiaotong University, China. The canines were randomly divided into two groups. Animals in the M-OLT group received M-OLT. Total hepatectomy of the recipients was performed at the time of delivery, and the suprahepatic inferior vena cava (SHIVC), infrahepatic inferior vena cava (IHIVC), and portal vein (PV) were reconstructed using the cuff technique. In the control group, the recipients underwent classic OLT without venovenous bypass (VVB). Liver graft revascularization was performed using the traditional hand-sewn vascular anastomosis technique. All liver transplantation procedures were performed by an experienced liver transplant surgeon (The surgeon has completed over 300 clinical liver transplant surgeries). The dogs were randomly selected as recipients and donors in each group, fasted but free to water for 8 h before surgery. All animals underwent propofol-induced general anesthesia before intubation (4–6 mg/kg IV) and were maintained on isoflurane in oxygen.

This study was approved by the Ethics Committee of Animal Experiments at Xi'an Jiaotong University (Permit number: XJTUAE2023-2152). This study complied with the ARRIVE guidelines and the Guide for the Care and Use of Laboratory Animals (8th, 2011).

### Design of total hepatectomy and liver graft implantation in the M-OLT group

**Fig. 1:**
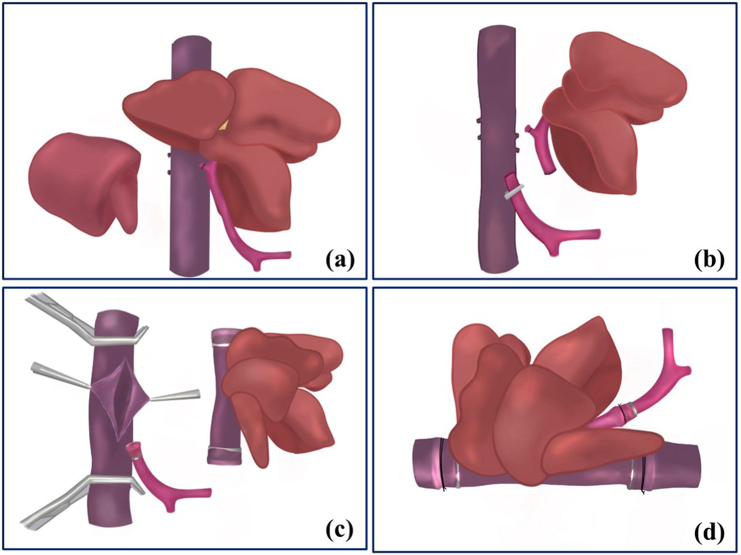
Design of total hepatectomy and liver graft implantation in the M-OLT group. (a) right lateral lobectomy without portal vein occlusion; (b) left lateral lobectomy with preservation of the IVC after portal vein occlusion; (c) Open notch on the side wall of the IVC; (d) reconstruction of the SHIVC, IHIVC, and PV using the cuff technique.

Total hepatectomy was completed in stages based on the anatomical structure of the canine liver. First, the hepatic pedicle of the right lateral lobe was isolated and ligated without occlusion of hepatic inflow. The hepatic vein trunk of the right lateral lobe was then dissected from the surrounding tissues, ligated, and disconnected. The right lateral lobe was then removed (Fig. 1a). The median and caudate lobes were excised using the same technique. Finally, the left lateral lobe was removed after portal vein occlusion while preserving the entire IVC (Fig. 1b).

Liver graft implantation was performed by opening the side wall of the IVC after vascular clamps were placed on the SHIVC and IHIVC (Fig. 1c). The cuffed donors, the SHIVC and IHIVC, were inserted into the recipient SHIVC and IHIVC from the opening of the IVC. The anastomosis was completed using a circumferential 7# silk suture. The PV reconstruction was completed using a circumferential 7# silk suture after the cuffed donor PV was inserted into the recipient PV (Fig. 1d). The PV, SHIVC, and IHIVC clamps were removed. The liver graft was recirculated and the anhepatic phase ended.

### Liver graft preparation

**Fig. 2:**
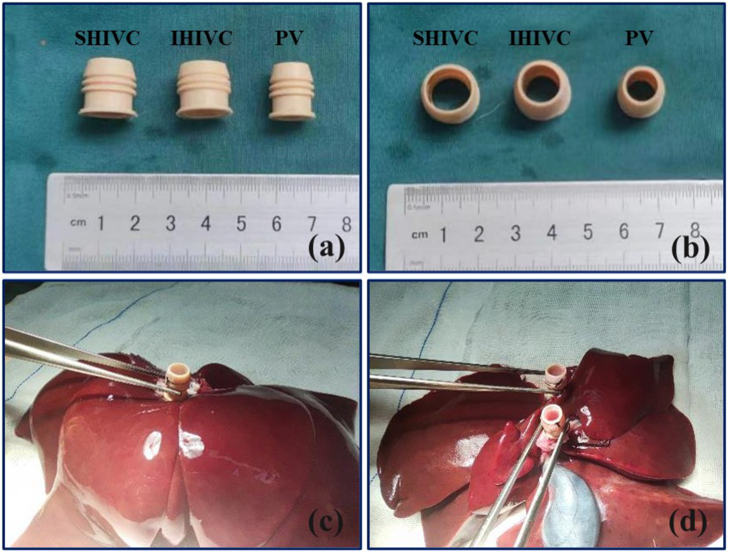
Liver graft preparation. (a–b) The cuff body with two grooves on the surface for the SHIVC, IHIVC, and PV; (c) the cuff is attached in the SHIVC; (d) the cuffs are attached in the IHIVC and PV.

The cuff segments for the SHIVC, IHIVC, and PV consisted of a 2-cm-long cuff body with two grooves on the outer surface (Fig. 2a). The external diameters of the cuff used for the SHIVC, IHIVC, and PV were 1.2 cm, 1.2 cm, and 0.8 cm, respectively (Fig. 2b). The diaphragm and connective tissues were removed from the SHIVC, and the phrenic vein was ligated. The IHIVC, SHIVC, and PV of the liver grafts were dissociated by at least 3 cm. The cuff was held with forceps and another forceps was passed through the lumen to grasp the PV. The open end of the PV was spread using three forceps. The end of the PV was then everted over the body of the cuff and fixed using two circumferential 4# silk sutures.

### Liver graft implantation

**Fig. 3:**
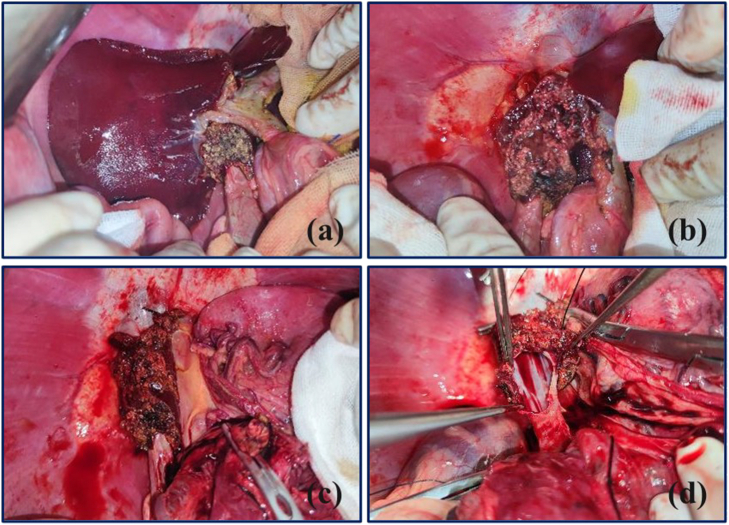
Total hepatectomy while preserving the entire IVC. (a) The right lateral lobule has been removed; (b) the median lobule has been removed without portal vein occlusion; (c) the left lateral lobule has been removed with preservation of the IVC after PV occlusion; (d) the lateral wall of the IVC is opened.

**Fig. 4:**
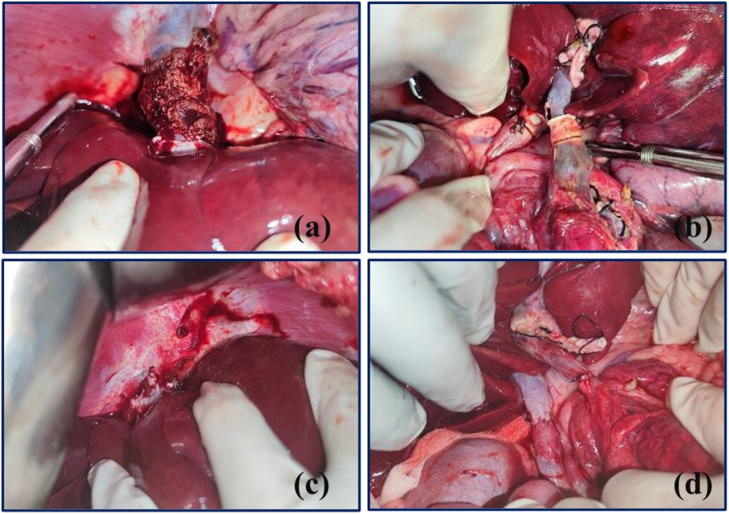
Liver graft implantation. (a) The SHIVC is connected by the cuff technique; (b) the IHIVC and PV are connected by the cuff technique; (c) reconstruction of the SHIVC by manual sutures; (d) reconstruction of the IHIVC and PV by manual sutures.

The recipient abdomen was incised through a J-shaped incision, and the hepatic hilum was dissected by separating the hepatic artery and the bile duct. Hepatic ligaments were removed, and the PV and IHIVC were fully freed by 3 cm. The hepatectomy was completed in stages (Fig. 3). The SHIVC, IHIVC, and PV anastomoses were completed using the cuff technique in the M-OLT group (Fig. 4), whereas the blood vessels were manually reconstructed using a 4-0 Prolene suture by manual suture in the control group. Finally, end-to-end hepatic artery anastomosis was performed using a 7-0 Prolene suture by manual suturing, and end-to-end biliary anastomosis was performed using a 5-0 absorbable suture by manual suturing.

The warm ischemic time (WIT) is defined as the rewarming time during the recipient's surgery from the start of vena cava anastomosis to portal vein reperfusion. The cold ischemic time (CIT) is defined as the time between perfusion with cold preservation solution and recovery of the liver graft blood inflow. The anhepatic phase was defined as the time from portal vein occlusion to graft recirculation. Operation time was defined as the time from the beginning of the skin incision to the end of the abdominal closure.

### Postoperative management

An intravenous drip of 3 mg/kg cefoperazone sulbactam sodium (Pfizer Inc., New York City, NY, USA) was administered daily for 72 h to prevent infection after liver transplantation. Intravenous fluids were administered for 3 days, after which oral hydration was permitted. The animals received intravenous injections of 1.0 mg/kg flurbiprofen axetil (Beijing Taide Pharmaceutical Co., Ltd., Beijing, China) twice daily within 1 week after surgery to relieve pain; Tacrolimus (0.2 mg·kg^−1^·d^−1^, Astellas Ireland Co. Ltd., Ireland) combined with mycophenolate mofetil (110 mg·kg^−1^·d^−1^, Roche R&D Center (China) Ltd., China) was used as the postoperative immunosuppressive regimen. Vascular ultrasound examination and transfemoral venography was performed immediately and one week after surgery to evaluate the patency of the vascular anastomotic stoma. We have been taking care of the surviving recipients liver transplantation, and we did not sacrifice them. However, euthanasia was performed by a 20 % solution of sodium pentobarbital injection via intravenous route as a bolus of 100 mg per kg body weight when the canines suffered from a non-recoverable state of health conditions such as major organ failure, nonresponsive medical conditions, anorexia and considerable loss of body weight due to illness, loss of consciousness or persistent irreversible hypothermia.

The WIT and CIT of the liver graft, operative time, intraoperative blood loss, anhepatic time, vascular occlusion time, and postoperative recipient death within one week were compared between the two groups.

### Statistical analysis

The continuous variable data are presented as median (interquartile range), while the categorical variable data are presented as n (%). Differences between means were assessed using the paired *t*-test, Wilcoxon signed-rank test, or chi-square test, where applicable. Statistical significance was established at *P* < 0.05. Statistical analysis was carried out using SPSS 21.0 (Chicago, IL, USA).

## Results

Liver transplantation was completed successfully in both groups, and none of the recipients died during surgery. There were no significant differences in the weight or sex of the donors and recipients between the two groups (all *P* > 0.05, see Table 1). The CIT of the liver graft was comparable between the two groups.

**Table 1:** Surgical information of dogs undergoing LT in two groups.

	M-OLT (*n* = 10)	Control (n = 10)	*p* Value
Donor information			
Weight, kg	16.5 (15.8–17.2)	17.0 (16.1–17.9)	0.24
Sex			1.00
Male	6	6	
Female	4	4	
CIT, hours	3.0 (2.8–3.0)	2.9 (2.8–3.0)	0.65
Recipient information			
Weight, kg	19.0 (18.8–20.3)	19.0 (16.8–20.0)	0.42
Sex			0.35
Male	4	5	
Female	6	5	
Operation time, minutes	120 (104–131)	155 (128–183)	<0.05
Intraoperative blood loss, mL	110 (98–120)	156 (145–170)	<0.05
Anhepatic time, minutes	10 (8–11)	33 (30–36)	<0.05
WIT, minutes	5 (4–5)	28 (27–30)	<0.05
Vascular occlusion time, minutes			
Portal vein	10 (8–11)	33 (30−33)	<0.05
Vena cava	7 (6–8)	30 (28–33)	<0.05
Postoperative death within 24 h	1	3	<0.05

CIT: cold ischemia time, WIT: warm ischemia time.

Recipients in the M-OLT group had a shorter operative time and less intraoperative blood loss (operative time: 120 (IQR = 104–131) min vs. 155 (IQR = 128–183) min, *P* < 0.05; intraoperative blood loss: 110 (IQR = 98–120) mL vs. 155 (IQR = 145–170) mL, *P* < 0.05) (Table 1). The anhepatic time in the M-OLT group was 10 (IQR = 8–11) min, which was significantly shorter than that of the control group (10 (IQR = 8–11) min vs. 33 (IQR = 30–36) min, *P* < 0.05, see Table 1). The WIT and occlusion time of the portal vein and vena cava were also significantly different between the two groups (WIT: 5 (IQR = 4–5) min vs. 28 (IQR = 27–30) min, P < 0.05; portal vein: 10 (IQR = 8–11) min vs. 33 (IQR = 30–36) min, P < 0.05; vena cava: 7 (IQR = 6–8) min vs. 30 (IQR = 28–33) min, P < 0.05) (Table 1).

**Fig. 5:**
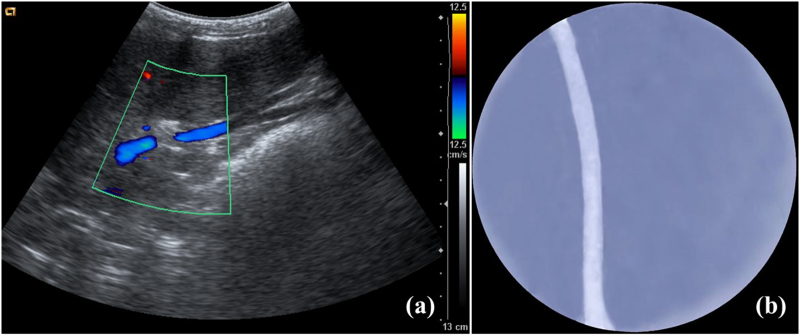
Representative imaging findings after surgery in the M-OLT group. (a) PV ultrasound image; (b) IVC angiographic image.

There was no stenosis or leakage of the portal vein or venae cavae anastomoses in either group immediately and 1 week after surgery (Fig. 5). One recipient from the M-OLT group died within 1 week after surgery due to arterial anastomotic bleeding, whereas three recipients in the control group died from respiratory failure (Table 1).

## Discussion

In this study, we introduced a modified OLT method using cuff technique for rapid liver graft implantation in the anhepatic period in canines, and the anhepatic time and WIT of the liver graft were significantly shortened.

Total hepatectomy and liver graft implantation are two critical steps that occur during the anhepatic period (AP) of liver transplantation. Unlike the piggyback technique, hepatectomy with preservation of the recipient's IVC in the M-OLT group is performed in stages in this study. This design is followed for the following reasons. First, each liver lobe has an independent inflow and drainage system, and the liver lobes are also independent of each other in canines ([12], [13], [14]). Therefore, the inflow and outflow vessels could be easily detected, ligated, and isolated. Second, the portal vein is not always occluded during a staged hepatectomy and can improve intestinal hemodynamic disorders.

The cuff technique, described by Kamada and Calne in 1975 (10), is a simple and fast method for IHIVC and PV anastomosis in rat liver transplantations. Subsequently, this technology has been applied to OLT in dogs and canines (11,15). However, this technology still has many shortcomings. First, at least three forceps are needed to tract the distal end of the vasculature and keep it open, making it difficult to operate in a narrow space, especially in laparoscopy. Additionally, the cuff may fall off the posterior wall of the vascular anastomosis due to the blind area in the surgical field of vision. However, the three major blood vessels were easily anastomosed using the cuff technique in this study, mainly because hepatectomy was performed with preservation of the recipient's IVC. The cuffed donor SHIVC and IHIVC were inserted into the recipient SHIVC and IHIVC, respectively, from the preserved vena cava notch, and the anastomosis was completed with a preplaced circumferential 7# silk suture. Only two forceps were required to open the venae cavae notch during this process, which means that this technique may be suitable for laparoscopic vascular reconstruction, especially for IVC anastomosis. In addition, the time required to complete this process was 7 [6–8] min, which was shorter than that required for traditional hand-sewn vascular anastomosis techniques. Furthermore, the cuff sleeves used in this study were made of polyetheretherketone generated by three-dimensional printing and exhibited good biocompatibility. The two grooves on the surface of the cuff prevented it from slipping from the vascular anastomosis. As a result, no anastomotic stenosis or leakage of the vena cava or portal vein occurred after liver transplantation.

Furthermore, a previous study indicated that a shorter graft revascularization is a protective factor against liver transplantation, particularly in the setting of graft marginality (8). However, revascularization of the liver is time-sensitive because minimizing the WIT and AP is critical for reducing allograft dysfunction, intestinal bacterial translocation, and acute liver failure ([16], [17], [18]). The SHIVC, IHIVC, and PV must be reconstructed during the AP using the classic OLT technique, and all vascular anastomoses are typically performed using the hand-sewn technique in clinical practice. Even among highly skilled surgeons, the duration of vascular suturing is one of the longest during the AP, especially in laparoscopic liver transplantation. Using the traditional suturing technique for major vascular reconstruction in laparoscopic liver transplantation will lead to the long operation time (19). Based on the result of this study, this modified technique may be a potential ideal method for liver implantation for laparoscopic liver transplantation.

After applying this modified liver transplantation technique, the anhepatic phase was shortened to approximately 10 [8–11] min in contrast to the 33 [30–36] min required for the traditional hand-sewn procedure. A previous study indicated that minimizing the AP is crucial for reducing postoperative mortality. In this study, the mortality rate of the M-OLT recipients within 24 h after transplantation was lower than that of the control group, which is consistent with previous findings (20).

This study had several limitations. First, the feasibility and safety of this novel technique in laparoscopic transplantation isn't verified in this study, but related research is currently ongoing. Second, the postoperative observation time was short (only 24 h), which did not allow an assessment of long-term outcomes. Third, compared with classic OLT techniques, the AP in this study was significantly shortened; thus, its impact on the postoperative liver and other organ functions remains unclear. However, previous studies have shown that shortening the anhepatic phase can improve liver graft ischemia-reperfusion injury (20).

In conclusion, this study successfully demonstrated the M-OLT technique to minimize the AP in canines. The promising results of the present study encourage future evaluation of this technique in human liver transplantation, especially for laparoscopic liver transplantation.

## Funding sources statement

This work was supported by the Key R & D plan of Shaanxi Province (No. 2021GXLH-Z-047) and Major Research Plan of the 10.13039/501100001809National Natural Science Foundation of China (92048202).

## Ethical approval statement

This study was approved by the Ethics Committee of Animal Experiments at Xi'an Jiaotong University (Permit number: XJTUAE2023-2152).

## CRediT authorship contribution statement

**Jie Hao:** Writing – original draft, Project administration, Conceptualization. **Jing-Wen Xiao:** Writing – original draft, Project administration. **Lin-Biao Xiang:** Project administration. **Rong Peng:** Project administration. **Jia-Qi Quan:** Project administration. **Ya-Xu Dong:** Project administration. **En-Hui Li:** Project administration. **Juan-Juan Wang:** Project administration. **Lu Ren:** Validation. **Yong Wan:** Validation. **Hong-Ke Zhang:** Project administration. **Yi Lv:** Supervision, Methodology, Funding acquisition, Conceptualization. **Qiang Lu:** Writing – review & editing, Supervision, Project administration, Methodology, Funding acquisition, Conceptualization.

## Declaration of competing interest

No authors have conflicts of interest or financial ties to disclose.
